# Galvanic synthesis of three-dimensional and hollow metallic nanostructures

**DOI:** 10.1186/1556-276X-9-679

**Published:** 2014-12-16

**Authors:** Sun Hwa Park, Jin Gyeong Son, Tae Geol Lee, Jongwon Kim, Sang Yun Han, Hyun Min Park, Jae Yong Song

**Affiliations:** Korea Research Institute of Standard and Science, Daejeon, 305-340 Republic of Korea; Department of Chemistry, Korea Advanced Institute of Science and Technology, Daejeon, 305-701 Republic of Korea; Department of Chemistry, Chungbuk National University, Chungbuk, 361-763 Republic of Korea; Department of Nanochemistry, Gachon University, Gyeonggi, 461-701 Republic of Korea; Korea University of Science and Technology, Daejeon, 305-350 Republic of Korea

**Keywords:** Nanoporous, Gold, Platinum, Palladium, Galvanic reaction

## Abstract

We report a low-cost, facile, and template-free electrochemical method of synthesizing three-dimensional (3D) hollow metallic nanostructures. The 3D nanoporous gold (3D-NPG) nanostructures were synthesized by a galvanic replacement reaction (GRR) using the different reduction potentials of silver and gold; hemispherical silver nanoislands were electrochemically deposited on cathodic substrates by a reverse-pulse potentiodynamic method without templates and then nanoporous gold layer replicated the shape of silver islands during the GRR process in an ultra-dilute electrolyte of gold(III) chloride trihydrate. Finally, the wet etching process of remaining silver resulted in the formation of 3D-NPG. During the GRR process, the application of bias voltage to the cathode decreased the porosity of 3D-NPG in the voltage range of 0.2 to -0.62 V. And the GRR process of silver nanoislands was also applicable to fabrication of the 3D hollow nanostructures of platinum and palladium. The 3D-NPG nanostructures were found to effectively enhance the SERS sensitivity of rhodamine 6G (R6G) molecules with a concentration up to 10^-8^ M.

**Electronic supplementary material:**

The online version of this article (doi:10.1186/1556-276X-9-679) contains supplementary material, which is available to authorized users.

## Background

Nanoporous gold (NPG) structures have received a great deal of attention due to their potential applications in the fields of double layer capacitors, fuel cells, biosensors, electrocatalysis, etc. [1–4]. Many researchers have studied nanoporous metallic materials with a large specific surface area (i.e., narrow pore-size distributions), because the nanoporosity affects not only active sites and electron mobility in the solid ligaments but also geometrical confinement effects [4–8]. It was reported that Pt-coated NPG nanostructures with smaller pore-size exhibited a higher catalytic activity in methanol oxidation than those with larger pore-size [9–11]. Recently, it was demonstrated that the surface enhanced Raman spectroscopy (SERS) effects of NPG depended on the pore size, the ratios of ligaments to nanopores, and the surface roughness [12, 13].

The typical methods of synthesizing NPG structures have been electrodeposition, anodization, and dealloying processes [14–16]. Generally, three-dimensional (3D) structures of NPG have been fabricated using the hard or soft templates of porous membranes and self-assembled spheres of dielectric/conductive materials [9–11, 17–20]. Recently, dual-templates of porous alumina and polystyrene microspheres were utilized to fabricate hierarchical macro/mesoporous gold wires [21]. Due to the combination of high specific surface area and easy transport of reactants in an electrochemical system, the 3D structures of modified NPG have attracted growing interest for applications such as electrocatalysis, energy conversion, and energy storage [5, 22]. Among these structures, 3D hollow structures of NPG have attracted more attention due to their high surface area, low density, usable nanoscale inner space, and unusual characteristics determined by shape and composition [23, 24]. However, the template-based process of fabricating 3D nanostructures is still complicated and time-consuming. More recently, for the low-cost mass production of NPG structures, Jiao et al. reported the patterned NPG two-dimensional array was produced by a straightforward imprinting process [25].

Here, we have developed a facile and low-cost electrochemical method of fabricating 3D hollow nanostructures of nanoporous gold, based on the filamentary deposition and galvanic reduction reaction without templates or surfactants [26]. The present method was able to be applied to fabricate 3D hollow nanostructures of gold, platinum, and palladium. And it is demonstrated that the 3D nanoporous gold (3D-NPG) nanostructures, as uniform SERS substrate without hot spots, exhibit a higher SERS enhancement factor than planar nanoporous gold films.

## Methods

### Fabrication of 3D hollow nanostructures

The silver nanoislands were electrodeposited in an electrolyte of 20 μM AgNO_3_ (#209139, reagent A.C.S., Sigma-Aldrich, St. Louis, MO, USA) and 2.11 mM NH_4_OH (#13370-0380, guaranteed reagent, Junsei, Tokyo, Japan) using a reverse-pulse potentiodynamic electrochemical method [26]. The reverse-pulse potentiodynamic process was performed at a reduction potential (V_R_) of 14 V and oxidation potential (V_O_) of 0.5 V for 2 h at the frequency of 0.5 Hz using the electrochemical system (Solartron, Model 1280z). Three electrodes were used; a Pt wire (0.5-mm in diameter and 1-m in length, Sigma-Aldrich) as a counter electrode, a KCl-saturated Ag/AgCl electrode as a reference electrode, and a sputtered Au film (90 nm in thickness) on a Si wafer as a working electrode. Galvanic replacement reaction (GRR) of the silver nanoislands was performed in an elect rolyte of 50 μM HAuCl_4_ · nH_2_O (*n* = 3.5, Kojima Chemicals Co., #903060) for 10, 24, 48, and 72 h without stirring. The GRR process resulted in the formation of core/shell structures, i.e., silver core and gold shell, because the silver atoms on the hemispherical silver islands were replaced by gold atoms. Then, a selective etching process of the silver core was performed in a 7.5 M HNO_3_ solution. And a process was carried out in a NH_4_OH solution (28 to 30 vol. %) to remove AgCl precipitates formed during the GRR process. The bias voltages of 0.2, -0.3, and -0.62 V was applied between the cathodic specimen and anodic Pt wire during the GRR process for 24 h in order to control the porosity of 3D-NPG nanostructures.

Three-dimensional platinum nanostructures with a nanoporosity were synthesized by the subsequent GRR process of the same silver nanoislands in the electrolyte of 50 μM H_2_PtCl_6_ · H_2_O (#254029, 99.995% trace metals basis, Sigma-Aldrich) while three-dimensional palladium nanostructures were produced by the GRR process in the electrolyte of 100 μM Na_2_PdCl_4_ (#379808, 99.99% trace metals basis, Sigma-Aldrich) at the bias voltage of -0.6 V.

Planar nanoporous gold (PNPG) films were synthesized on silver films (60 nm in thickness) which were sputter-deposited on Au coated Si substrates. The GRR process was performed in an electrolyte of 50 μM HAuCl_4_ · nH_2_O for 12 h without a bias voltage. And then the selective etching process of sliver atoms led to the formation of the PNPG films.

### Characterization

The morphologies and crystal structures of the 3D nanostructures were analyzed by a field-emission scanning electron microscope (SEM; Hitachi S-4800, Hitachi Ltd, Chiyoda-ku, Japan) and a high resolution transmission electron microscope (HRTEM; FEI Tecnai G^2^ F30, 300 kV, FEI, Minato-ku, Japan) equipped with an energy dispersive X-ray spectroscopy detector (EDS; EDAX Inc., Kanagawa, Japan), respectively. The relative electrochemical surface areas (rESA) of the nanostructures were evaluated by measuring the charge quantity consumed for the reduction of the surface oxide layer using a cyclic voltammogram at a scan rate of 50 mV∙s^-1^ in a N_2_-saturated electrolyte of 0.1 M H_2_SO_4_. The SERS measurements were performed using a homemade micro-Raman system based on a 633 nm He/Ne laser and a 100 × objective lens with a thermoelectrically cooled CCD detector (iDUS 401, ANDOR, Belfast, Northern Ireland, UK). The laser power was approximately 0.25 mW and the focused spot size was 1 μm. The spectra were obtained at random locations for each sample with an integration time of 10 s for all the measurements. In order to conduct SERS measurements, the nanostructures on substrates were immersed in a rhodamine 6G (R6G) aqueous solution with a concentration in the range of 10^-4^ to 10^-8^ M, rinsed thoroughly with high purity water for the formation of mono-dispersed R6G molecules, and then dried under blowing N_2_.

## Results and discussion

### 3D nanoporous gold structures

**Figure 1:**
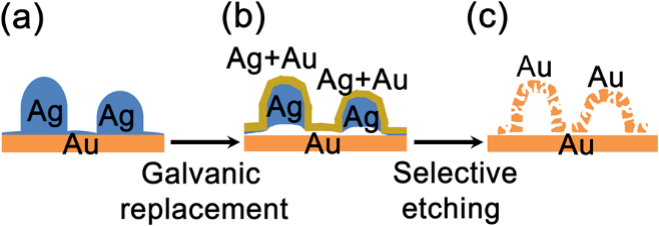
**Schematic diagram depicting the three fabrication steps of 3D-NPG nanostructures. (a)** Hemispherical silver nanoislands, **(b)** core-shell nanostructures after GRR, and **(c)** 3D-NPG nanostructures after a selective etching of silver.

Figure 1 shows a schematic diagram that the 3D-NPG ultra-thin nanostructures were formed by the GRR and selective etching processes. First, hemispherical silver nanoislands are electrodeposited on a cathodic Au substrate without any templates or surfactants, as reported in a previous study (Figure 1a) [26]. Secondly, the silver atoms on the nanostructure surface are replaced by gold atoms, according to the GRR process, when the nanostructure is immersed in a 50 μM HAuCl_4_ · nH_2_O aqueous solution without any external voltages, as shown in Figure 1b. The 3D-NPG nanostructures are formed by replicating the silver nanoislands, after a selective etching of the silver cores (Figure 1c).

**Figure 2:**
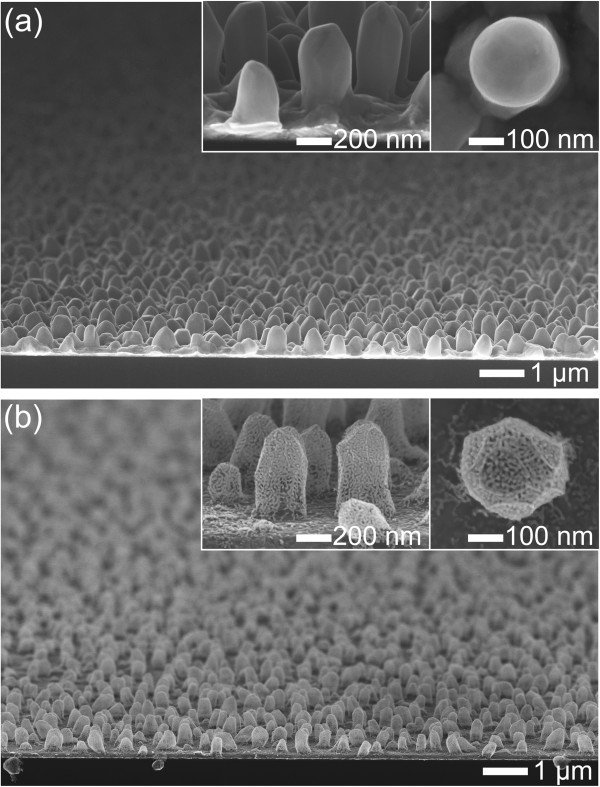
**Tilted SEM images of (a) hemispherical silver nanoislands and (b) 3D-NPG nanostructures.** After a GRR process for 24 h and silver etching process. The insets denote tilted and top-view SEM images with a higher magnification, respectively.

Figure 2a shows typical SEM images of the hemispherical silver islands (300 ± 28 nm in diameter) deposited on the Au-coated Si substrate. The silver nanoislands, which had an aspect ratio of approximately 2, were uniformly distributed over the entire substrate. Figure 2b exhibits the typical 3D-NPG nanostructures formed by the subsequent processes of GRR and selective etching. The GRR process occurred according to the following reaction of Equation 1 [20],

1

AuCl_4_^-^ is reduced to one Au atom and silver is oxidized to form AgCl. The GRR generates the small pits on silver nanoislands (marked by the yellow arrows in Additional file 1: Figure S1a), which have relatively high surface energy [20, 27]. During the GRR process, gold ions are expected to be reduced forming facetted surfaces because gold and silver phases have the same face-centered crystal structure, with lattice constants of 4.0786 and 4.0862 Å, respectively [20, 27]. Thus, a thin Au facetted layer is formed replicating the surface of silver nanoislands and prevents underlying silver from reacting with AuCl_4_^-^ ion.

The etching process occurs through the small pits, which play a role of the diffusion pathway for Ag^+^ (as indicated by arrows in Additional file 1: Figure S1a-d). During the etching process, silver in the core and silver existing in the thin Au shell layer are selectively dissolved and resulted in the formation of hollow and nanoporous Au shell structure, i.e., 3D nanoporous gold structure. The nanoporosity might be ascribed that AuCl_4_^-^ is reduced to one gold atom at the expense of three silver atoms, according to Equation 1. As the surface area and surface energy increase with further etching of silver, the reconstruction of the pore morphology occurs via Ostwald ripening process [27, 28].

**Figure 3:**
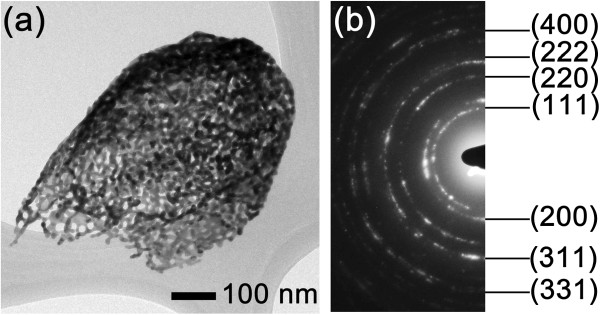
**TEM images of the 3D-NPG nanostructure. (a)** BFTEM image and **(b)** SAED pattern.

GRR in ultra-dilute electrolyte led to the formation of uniform and ultra-thin 3D-NPG nanostructures because the low concentration of the electrolyte decreased the concentration gradient between the cathodic surface and solution and formed a thick double layer (diffusive region) leading to a slow reaction-rate of galvanic replacement [26, 29, 30]. Therefore, for the ultra-dilute electrolyte (50 μM), the GRR occurred only on the surface of the silver islands and after 24 h resulted in the formation of the isolated 3D-NPG on the substrate (see yellow dotted lines in Additional file 1: Figure S2a). With further time up to 48 h, the GRR occurred over the entire bottom surface as well as the silver nanoislands, forming the interconnected 3D-NPG (see Additional file 1: Figure S2b). However, with the further GRR time up to 72 h, the nanopores of 3D-NPG gradually disappeared, and thick gold walls with smooth surfaces were produced by oxidizing silver in the core (Additional file 1: Figures S1d and S1h). In contrast, when the electrolyte concentration was increased to 200 μM HAuCl_4_ · nH_2_O, the initial hemispherical shape was not maintained and became rough because the GRR occurred faster and severely (Additional file 1: Figure S3). In addition, AgCl precipitates substantially formed on the nanostructure surface (see the yellow arrows marked in Additional file 1: Figure S3).Figure 3 shows the typical bright field TEM (BFTEM) image and selected area electron diffraction (SAED) pattern of 3D-NPG shown in Figure 2b. The SAED shows a ring-like pattern indicating that the 3D-NPG has a face-centered-cubic polycrystalline structure. The ring patterns are indexed to be (111), (200), (220), (311), (222), (400), and (331) reflection planes from the center, in sequence. And it is noted that the 3D-NPG is composed of many nanopores (less than several tens of nanometers in diameter).

### 3D-nanoshell gold structures

**Figure 4:**
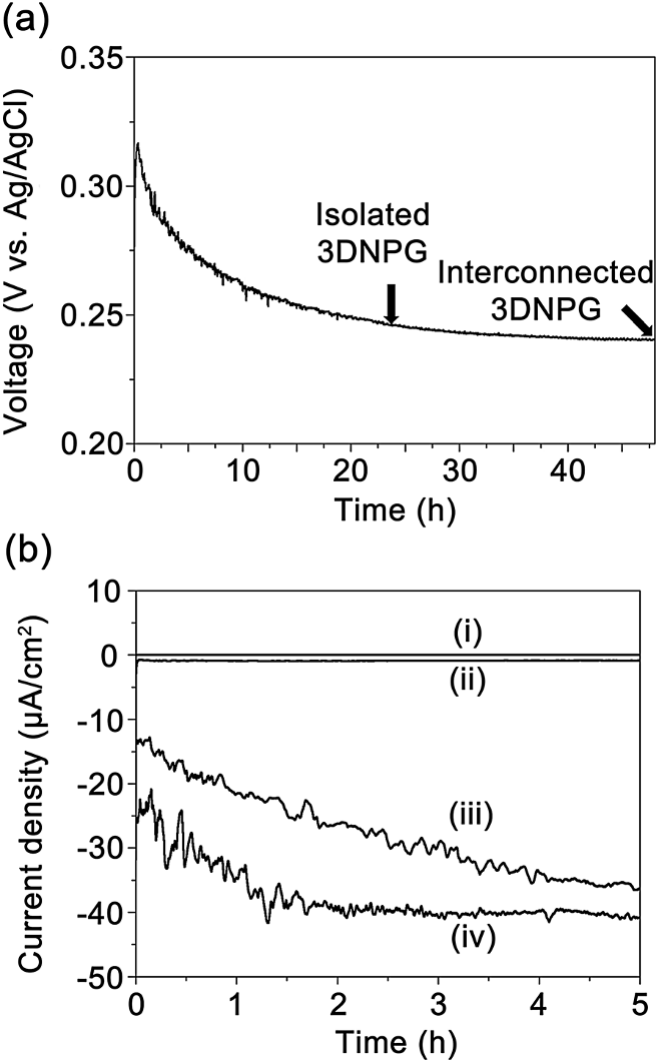
**Variation of open circuit voltage (OCV) and current density. (a)** Variation of OCV during GRR process in a 50 μM HAuCl_4_ · nH_2_O electrolyte and **(b)** variations of current densities with the bias voltage (V) of (i) OCV, (ii) 0.2, (iii) -0.3, and (iv) -0.64 during the GRR process, respectively.

**Figure 5:**
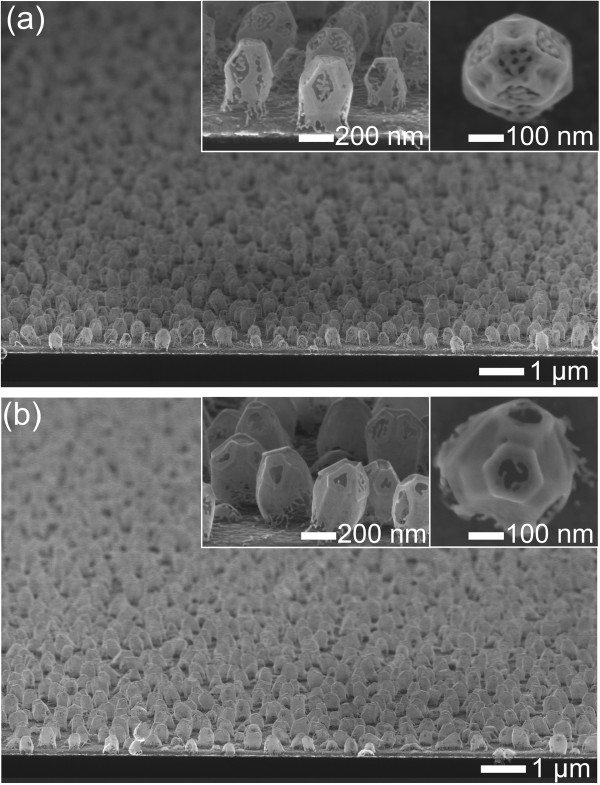
**Tilted SEM images of 3D-NPG nanostructures.** 3D-NPG nanostructures at the bias voltage of **(a)** -0.3 and **(b)** -0.62 V after the GRR process for 24 h and silver etching. The insets denote the tilted and top-view SEM images with a higher magnification, respectively.

As shown in the open circuit voltage variation with the GRR time up to 48 h (see Figure 4a), the voltage slowly decreased from 0.32 to 0.24 V. This suggests that the GRR might be controlled by applying an external bias voltage to the cathodic substrate. When the bias voltage was 0.2 V, the porosity of the final nanostructure was almost identical to that of 3D-NPG nanostructure, and the formation of AgCl was reduced (Figures 4b-ii and S4). Accordingly, the porosity of the 3D-NPG was controlled by applying the bias voltage in the range of 0.2 to -0.62 V. With the increase of bias voltage, the current density increased, as shown in Figure 4b. The increase of current density implied more supply of electrons which are needed for the reduction of AuCl_4_^-^ ions. Figure 5 shows that the increase of bias voltage decreased porosity and resulted in the formation of nanoshell gold structures. It is presumed that the bias voltage plays a role of supplying electrons and reducing the expense of silver atoms during the GRR process because AuCl_4_^-^ ions are reduced to one gold atom at the expense of three electrons supplied by silver, according to Equation 1. Thus, the consumption of silver atoms decreases, and then the porosity decreases when electrons are externally provided. This process is not only affected by the chemical reaction for galvanic replacement, but also the underpotential deposition of a metallic adlayer onto a different metallic substrate [30].

### 3D-nanostructures of platinum and palladium

**Figure 6:**
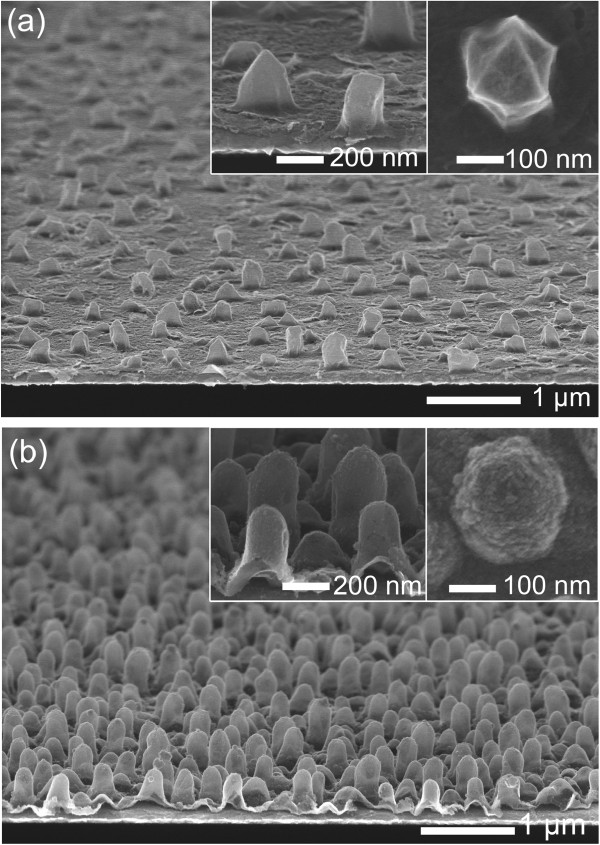
**Tilted SEM images of 3D nanoporous platinum and 3D-nanoshell palladium nanostructures.** The insets denote the tilted and top-view SEM images with a higher magnification, respectively.

The GRR process was utilized to synthesize 3D nanoporous platinum and 3D-nanoshell palladium structures, as shown in Figure 6. Figure 6a shows the typical SEM images of ultra-thin 3D nanoporous platinum nanostructures produced by the GRR process in a 50 μM H_2_PtCl_6_ · xH_2_O solution and a selective etching process. The redox reaction of silver and PtCl_6_^2-^ occurs following Equation 2 [24]. The 3D nanoporous platinum structures had smaller nanopores and face-centered cubic crystal structure, as indexed by TEM and electron diffraction pattern analyses (Additional file 1: Figure S5).

2

With a similar vein, 3D-nanoshell palladium nanostructures were fabricated by the GRR process in 100 μM Na_2_PdCl_4_ (bias voltage of -0.6 V) and a selective etching process, as shown in Figure 6b. It was noted that the 3D-nanoshell palladium structures had thick shells rather than porous structure with the help of bias voltage, as discussed above.

### SERS of 3D nanoporous gold structures

**Figure 7:**
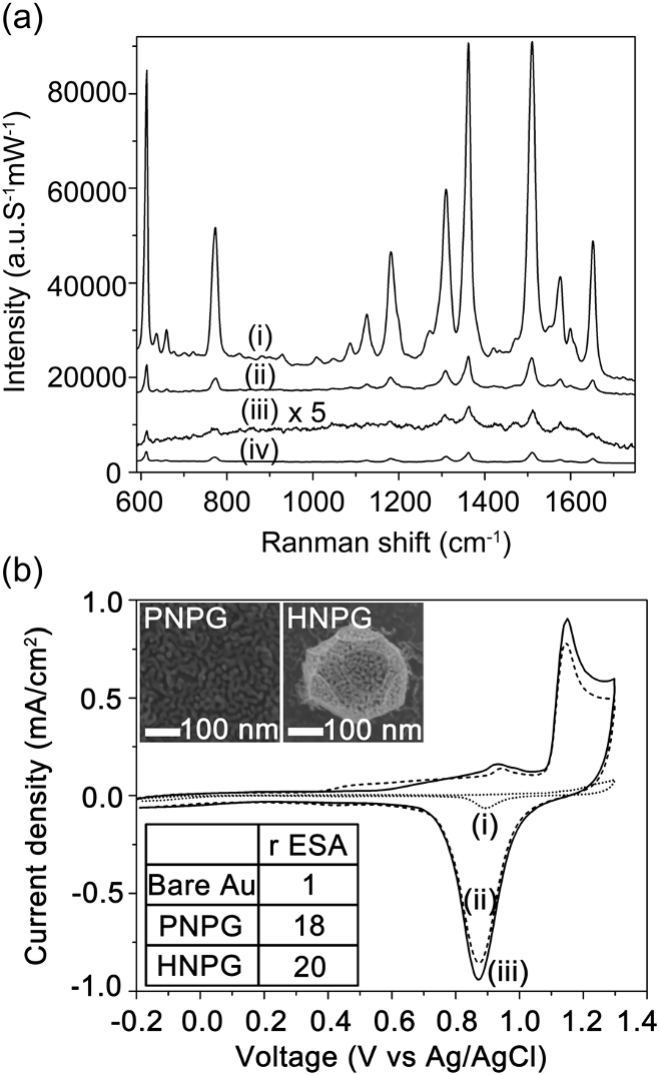
**Variation of SERS spectra with the R6G concentrations and cyclic voltammograms. (a)** SERS spectra with the R6G concentrations of (i) 10^-6^, (ii) 10^-7^, and (iii) 10^-8^ M on 3D-NPG nanostructures and (iv) 10^-6^ M on PNPG film, respectively. **(b)** Cyclic voltammograms measured for (i) bare Au film, (ii) PNPG film, and (iii) 3D-NPG nanostructures in N_2_-saturated 0.1 M H_2_SO_4_ for the sake of measuring each rESA. The insets denote top-view SEM images of PNPG and 3D-NPG, respectively.

Figure 7a shows the SERS spectra of R6G adsorbed on the 3D-NPG nanostructures and PNPG film with the R6G concentration in the range of 10^-6^ to 10^-8^ M. All the SERS peaks for 3D-NPG nanostructures are clearly distinguished by 633 nm laser and assigned to the characteristics of R6G Raman spectra [31, 32]. In comparison, the SERS intensity of R6G on PNPG film is approximately 40 times lower than that on 3D-NPG nanostructures, even though the surface areas of both nanostructures are almost similar to each other, according to rESA measurement (the below inset of Figure 7b). The rESA was evaluated by cyclic voltammograms in N_2_-saturated 0.1 M H_2_SO_4_, as shown in Figure 7b. The similar surface areas of 3D-NPG and PNPG are due to the less formation of nanopores around 3D hollow nanostructures, as shown in the inset of Figure 7b. Generally, the higher SERS intensity is known to come from nanpore size, ratios of ligaments to nanopores, and surface roughness [12, 13]. However, in the present study, the difference of pore sizes between 3D-NPG and PNPG was not so large, approximately 3 nm. This suggests that the SERS enhancement for 3D-NPG nanostructures might be due to other effects. One possible reason is supposed to be the polygonal edges of 3D-NPG (see the inset of Figure 7b). Previously, it was reported that high index gold nanocrystals exhibit more efficient SERS activity than spherical gold nanocrystals and an electromagnetic field enhancement effect arises because the electric field localizes more easily at the edges of nanocrystals [33]. And, the SERS spectra of 10^-6^ M R6G were measured at random points over the whole substrate. The SERS enhancement was highly reproducible at random five points over the whole substrate, as shown in the SERS spectra (Additional file 1: Figure S6). Thus, ultra-thin 3D-NPG nanostructures were easily fabricated by the bottom-up process providing a uniform SERS substrate without hot spots.

## Conclusions

We suggest a facile and low-cost process of fabricating 3D hollow metallic nanostructures. The 3D hollow nanostructures of gold, platinum, and palladium were uniformly synthesized using sacrificial silver nanostructures on a substrate. As the porosity of the 3D hollow nanostructures was able to be controlled by the bias voltage between the cathode and the anode, the nanoporous and nanoshell structures could be alternatively synthesized. In comparison with PNPG nanostructures, the 3D-NPG nanostructures were proved to be a superior SERS substrate with a higher enhancement of SERS intensity. The template-free electrodeposition and GRR process based on ultra-dilute electrolytes are expected to be utilized for further development of various 3D hollow porous nanostructures.

## Electronic supplementary material

Additional file 1: **SEM and TEM images.** SEM and TEM images of 3D nanostructures including SERS spectra for R6G molecules. (DOCX 1 MB)
