# Supplementary material for: Galvanic synthesis of three-dimensional and hollow metallic nanostructures
Source: Nanoscale Res Lett. 2014 Dec 16;9:679. doi: 10.1186/1556-276X-9-679 (PMC4493846; doi:10.1186/1556-276X-9-679)
Supplement: Supplementary file 1 — Additional file 1: SEM and TEM images. SEM and TEM images of 3D nanostructures including SERS spectra for R6G molecules. (DOCX 1 MB) [file 11671_2014_2403_MOESM1_ESM.docx]

**Electronic Supporting Information**

**Title: Galvanic synthesis of three-dimensional and hollow metallic nanostructures**

Sun Hwa Park, Jin Gyeong Son, Tae Geol Lee, Jongwon Kim, San Yun Han, Hyun Min Park and Jae Yong Song


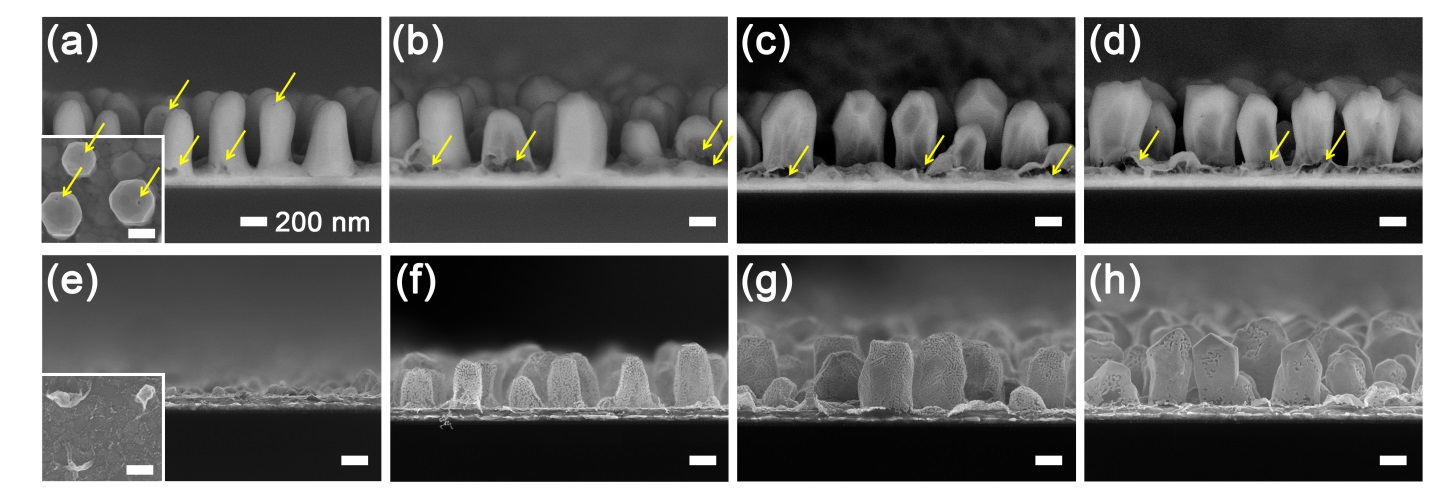


Fig. S1. Cross-sectional SEM images of silver nanoislands with GRR time of (a) 10, (b) 24, (c) 48 and (d) 72 hour in a 50 µM HAuCl_4_·nH_2_O electrolyte, respectively. (e-h) Cross-sectional SEM images of 3D-NPG after the selective etching of silver in nanostructures shown in (a) to (d), respectively. The yellow arrows indicate pits and voids formed at the top surface and bottom of 3D-NPG nanostructures. All the scale bars denote 200 nm.


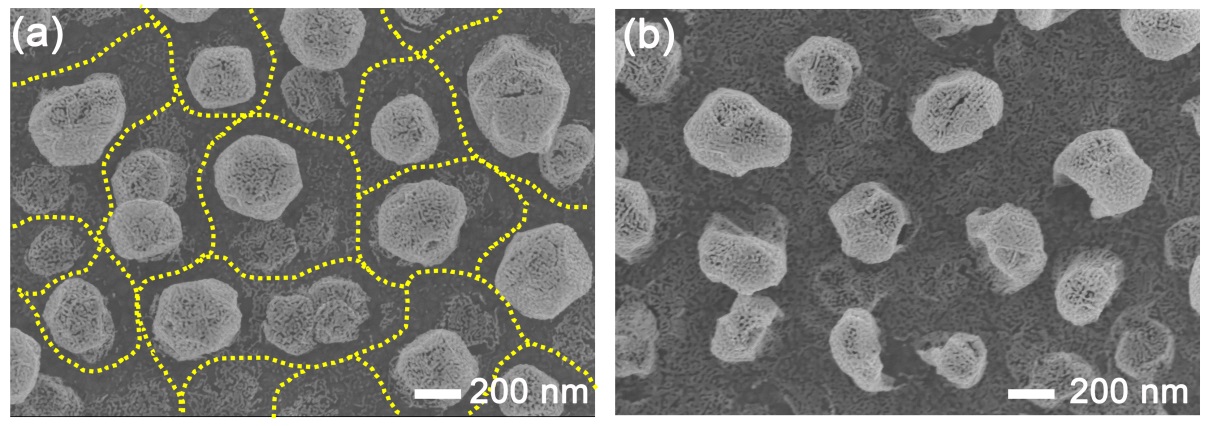


Fig. S2. Top-view SEM images of (a) isolated and (b) interconnected 3D-NPG nanostructures after the GRR process for 24 and 48 hours and selective etching process, respectively. The yellow dotted lines indicate that the 3D-NPG nanostructures are disconnected each other.


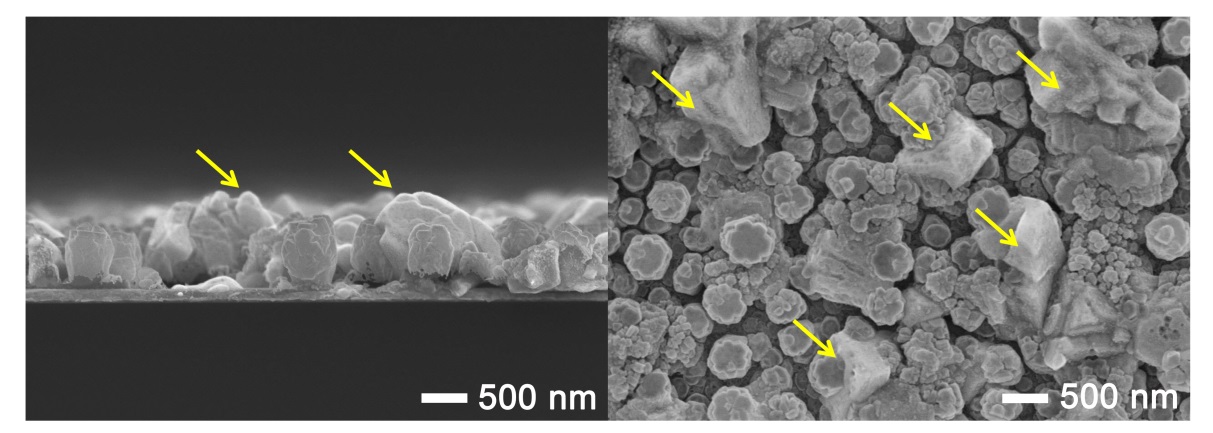


Fig. S3. Top-view and cross-sectional SEM images of the nanostructures after the GRR process in 200 µM HAuCl_4_· nH_2_O for 90 minutes. The yellow arrows indicate AgCl precipitates.


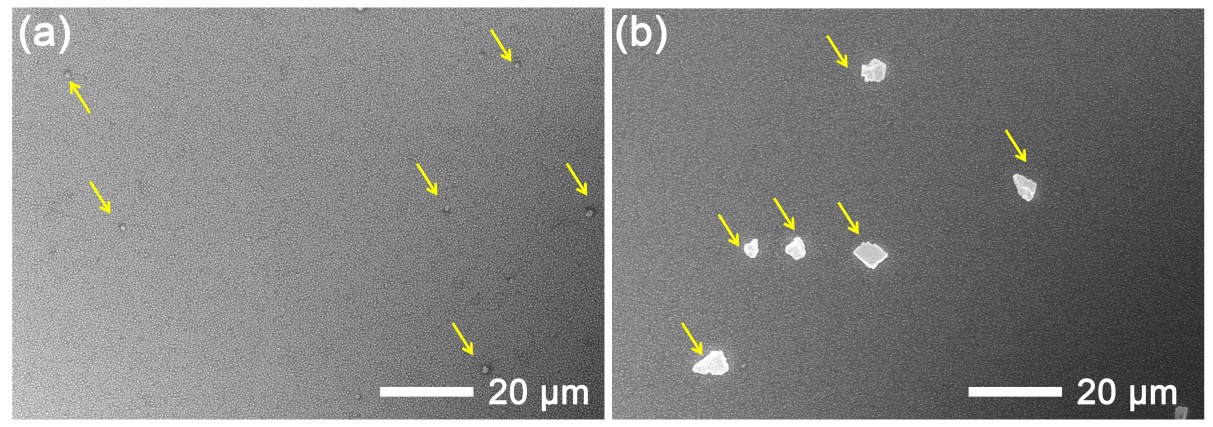


Fig. S4. Top-view SEM images of 3D-NPG nanostructures synthesized at (a) the bias voltage of 0.2 V and (b) without a bias voltage. The yellow arrows indicate the AgCl precipitates.


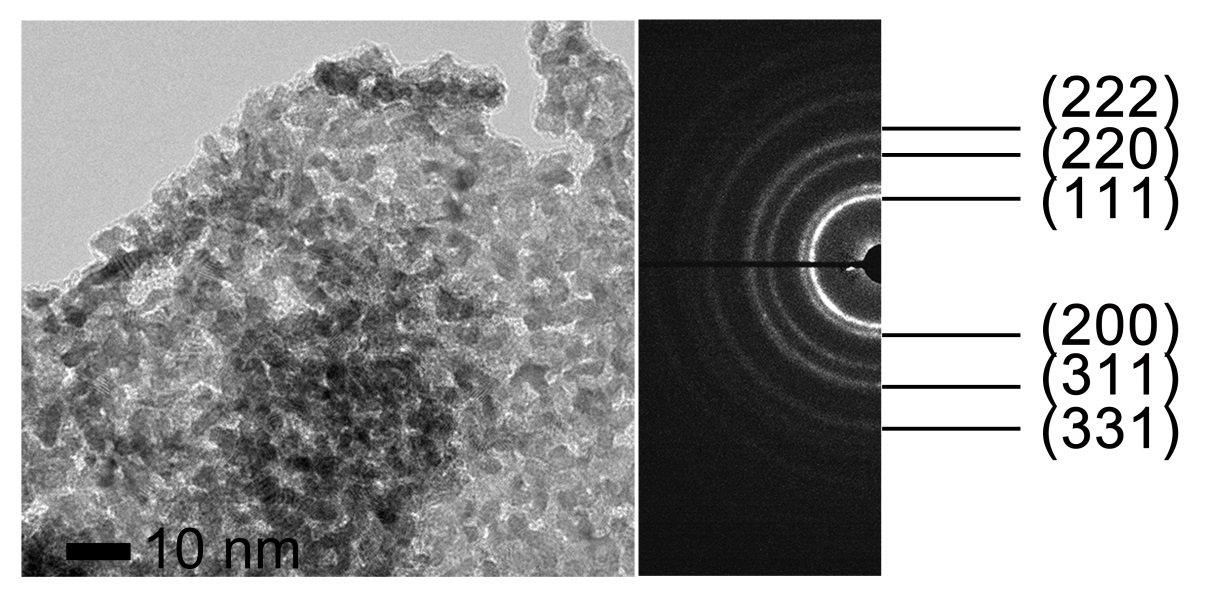


Fig. S5. (a) BFTEM image and (b) SAED pattern of the 3D-nanoporous platinum nanostructure.


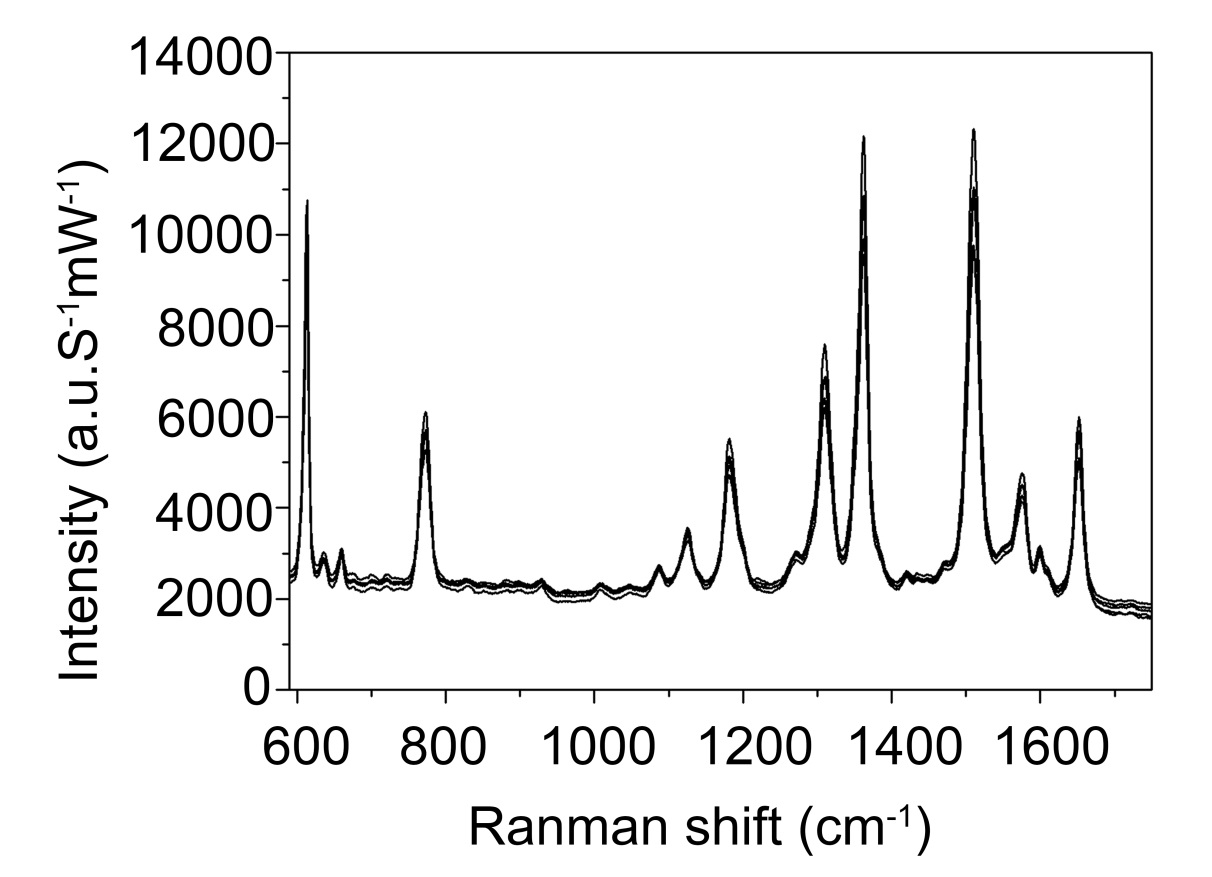


Fig. S6. Variations of SERS spectra of 10^-6^ M R6G for different position of 3D-NPG substrate.
